# Patient satisfaction in tele-ophthalmology: systematic review and meta-analysis

**DOI:** 10.1038/s41433-026-04831-4

**Published:** 2026-09-07

**Authors:** Khaldoon O. Al-Nosairy, Francie Stolle, Lars Choritz, Giulia Renieri, Tobias Duncker, Bernt-Peter Robra, Hagen Thieme

**Affiliations:** 1 Ophthalmology Department, Faculty of Medicine, Otto-von-Guericke University, Magdeburg, Germany; 2 Ophthalmology Institute, Halle, Germany; 3 Social Medicine and Health Services Research, Faculty of Medicine, Otto-von-Guericke University, Magdeburg, Germany

**Keywords:** Health care, Medical research

## Abstract

Telemedicine is rapidly expanding in eye care, yet evidence on patient satisfaction and perceived quality is limited, restricting wider clinical adoption and integration. This study aimed to: i) compare satisfaction rates in tele-ophthalmology versus in-person eye care; ii) assess overall patient satisfaction within tele-ophthalmology services. This systematic review was prospectively registered in PROSPERO (International Prospective Register of Systematic Reviews ID: CRD420251218938). We used random-effects meta-analyses to pool comparative satisfaction odds ratios (tele-ophthalmology vs. in-person care) and to calculate overall tele-ophthalmology satisfaction proportions. Heterogeneity was quantified using the *I*^2^ statistic. We included 41 studies encompassing 24,595 participants. Five studies reporting patient satisfaction in telemedicine vs in-person revealed comparable satisfaction rates (OR = 0.98; 95% CI = 0.36; 2.65, *p* = 0.95). Across all telemedicine studies (n = 36), the pooled satisfaction rate was high 93.5% (95% CI = 90.8, 95.5) with comparable rates across different tele-ophthalmology models [(${\chi }^{2}=0.99,{df}=2,{p}=0.61$), asynchronous (94.6%), synchronous (92.1%) and hybrid (93.8%)]. While general acceptability for telemedicine was high 92.0%, participants were more likely to recommend telemedicine service (94.9%) than to reuse it in the future (84.0%). No publication bias was found (*p* > 0.05). Patient satisfaction in tele-ophthalmology is high and appears to be comparable to standard in-person care. However, considerable variability between studies highlights the need for validated and standardised measures for patient satisfaction. Furthermore, lower intention rates for future use underscore the need to address potential systemic gaps, such as digital illiteracy, to sustain long-term adoption.

## Introduction

Preserving visual health is an essential pillar for overall wellness and serves as a key driver for sustainable development of societies [1]. As of 2020, more than 1.1 billion people were living with distance or near visual impairments [2] and this number projected to increase significantly over the next three decades driven by population aging and a higher prevalence of chronic eye diseases [2, 3]. This surge in demand for eye care is exacerbated by a global shortage of ophthalmologists highlighting the need for more scalable and efficient models of eye care delivery.

In the current era of digitalisation, telemedicine has the potential to address these unmet needs in ophthalmology practice. Tele-ophthalmology has been rapidly evolving from niche application to a mainstream model of eye care delivery particularly following the COVID-19 pandemic. It encompasses three broad categories: i) synchronous teleophthalmology using real-time video or telephone consultations; ii) asynchronous models (store-and-forward) involving acquisition of remote data for later specialists review, including home monitoring such as telemetric intraocular pressure measurements [4]; and iii) hybrid approaches using both modalities. While tele-ophthalmology has been utilised in many specific eye diseases with established feasibility and diagnostic precision, its wider more general use is hampered by legal barriers, technical requirements [5] and -critically- patient acceptance.

Patient satisfaction is a critical determinant for the successful implementation of telemedicine programs. Beyond mere preference, satisfaction metrics could reflect key outcomes such as adherence, follow-up compliance, and overall health. In general, patients express high satisfaction with telemedicine due to several factors, e.g., increased accessibility, improved communication and low costs [6]. However, evidence on patient satisfaction in tele-ophthalmology remains fragmented by disparate methods and inconsistent reports, leaving a void in quantitative synthesis.

Thus, a systematic assessment of patient satisfaction is required to guide the adoption, optimisation, and integration of these services into standard clinical workflows. This systematic review and meta-analysis study aimed to: (i) compare tele-ophthalmology vs in-person eye care, and (ii) evaluate overall patient satisfaction across different telemedicine models.

## Methods

This systematic review and meta-analysis investigated patient satisfaction with tele-ophthalmology. This review was conducted in accordance with the Statement of Preferred Reporting Items for Systematic Reviews and Meta-Analyses (PRISMA) [7] and is registered with PROSPERO (International Prospective Register of Systematic Reviews, ID: CRD420251218938).

### Search strategy

A literature search was conducted from database inception to November 26th, 2025, without language restrictions, using the following databases: CLIB (The Cochrane Library), PubMed, SCI (Science Citation Index) and Scopus. Search terms were: (teleophthalmology OR telemedicine OR telehealth OR teleconsult* OR ”virtual care” OR ”remote consultation” OR ”online consultation” OR tele-eye) AND (ophthalmol* OR ”eye care” OR optometr*) AND (”patient satisfaction” OR satisf* OR recommend* OR ”patient experience” OR ”patient perception” OR acceptance OR feasibility).

### Study selection

**Fig. 1:**
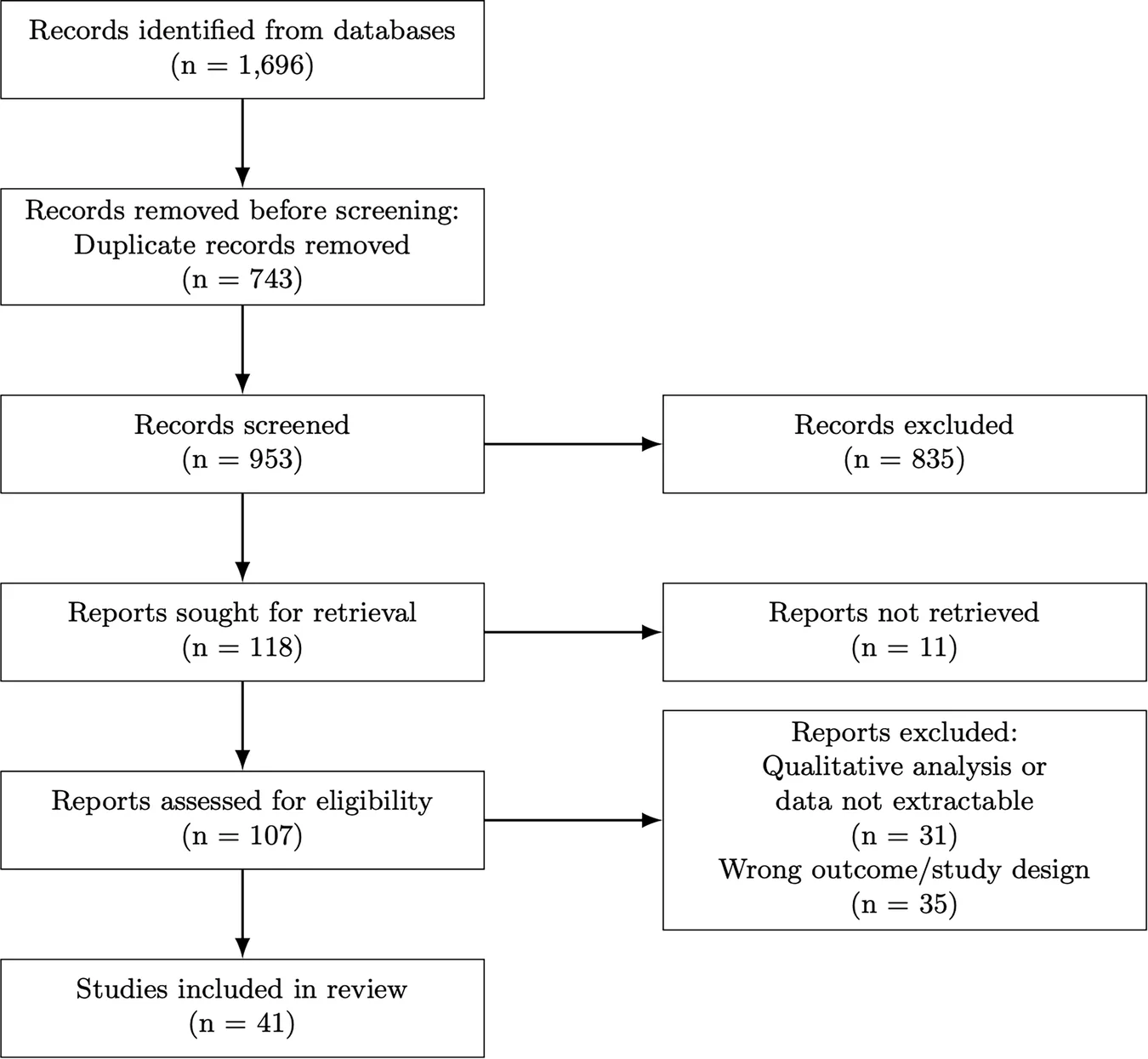
PRISMA flow diagram of study screening.

Studies were eligible for inclusion if a quantification metric for the of patient satisfaction was performed (or a comparable metric, e.g. perceived quality of care) with any form of tele-ophthalmology, either alone or compared to in-person care. Secondary outcomes included the willingness to recommend the care provided and the intention of reuse. Two authors (KAN and FS) independently screened retrieved records with the disagreements resolved by consensus. The authors screened 953 studies by title and abstract, excluding 835 studies. After full-text assessments for 118 studies, 41 were included in the final analysis (Fig. 1).

### Data extraction

Primary and secondary outcomes were extracted alongside demographics (age, sex), sample size and telemedicine modality, service settings (e.g., tertiary care or community services), eye subspeciality and whether it was meant for screening or follow-up.

Patient satisfaction outcomes reported as continuous or categorical responses were binarised (satisfied vs. not satisfied) according to individual study’s cutoffs.

### Risk of bias assessment

Methodological quality was assessed using the Joanna Briggs Institute (JBI) Critical Appraisal Checklists for non-randomised controlled studies and the Cochrane RoB2 or randomised controlled studies. Studies were rated as having low, moderate (some concerns) or high risk of bias based on the proportion of affirmative responses to the checklist items.

### Statistical analysis

Statistical analyses were performed in R (R Core Team, 2023). For comparative analysis, we used odds ratios (OR) as a summary measure. For single-arm tele-ophthalmology analysis, pooled proportions were estimated via generalised linear mixed models (GLMMs) to handle values approaching 0% or 100%. The raw proportions were transformed using a logit transformation for variance stability, and individual study confidence intervals (CI) were calculated using the Clopper–Pearson method.

Heterogeneity was assessed using the statistic *I*^2^ and Cochran Q test. An *I*^2^ value > 50% was considered indicative of substantial heterogeneity. Random effects models were used as the default analytical approach for all pooling given the expected variation in the context and settings of the included studies. Sub-group analysis was performed to explore sources of heterogeneity based on: 1) Technology modality (synchronous/video vs asynchronous/store-and-forward vs hybrid), 2) type of service provider/setting (Hospital, community, optometry, retmote/home), 3) patient status (new vs follow-up visits) and 4) tele-ophthalmology subspecialities (emergency vs glaucoma vs medical retina). A secondary separate analysis was performed for acceptability outcomes (willingness to recommend vs intention to reuse).

### Bias and robustness assessment

We used funnel plots and Egger’s linear regression test for asymmetry to assess publication bias alongside a leave-one-out sensitivity analysis to evaluate the robustness of the pooled estimates. A meta-regression was employed to assess trends in patient satisfaction over time.

## Results

We included 41 studies [8–48] (Fig. 1) comprising 24,595 participants. The majority of studies were based in the USA and the UK (Table 1). Studies were conducted mainly during or after the COVID19 pandemic, which marked a distinct shift in terms of the clinical context and settings. Prior to 2020, telemedicine focused largely on asynchronous diabetic retinopathy (DR) screening to enhance accessibility in many under-served regions. Thereafter, this shifted toward synchronous emergency tele-triage and virtual clinic models.

**Table 1:** Characteristics of included studies evaluating patient satisfaction with tele-ophthalmology.

Study	Country	Setting	Modality	Patient status	Subspecialty	*N* (Survey *n*)	Age [Years]	Female [%]
Wos et al. [8]	Poland	Hospital/Tertiary	Synchronous	New/Screening	Emergency	111	41.0 ± 14.2	78.4
Newman-Casey et al. [9]	USA	Community/Optometry	Asynchronous	New/Screening	Glaucoma	3740 (3407)	54.1 ± 15.9	62.5
Do et al. [10]^a^	USA	Remote/Home	Asynchronous	New/Screening	General	141 (101)	59.7 ± 14.0	60.4
Lim et al. [11]	Canada	Remote/Home	Asynchronous	New/Screening	Medical Retina	28 (19)	86.5	78.6
Organov et al. [12]	USA	Hospital/Tertiary	Synchronous	Mixed	General	520 (74)	34.9 ± 30.1	60.2
Dia et al. [13]^a^	USA	Hospital/Tertiary	Hybrid	Follow-up	General	49	65.0 (median)	59.2
Dole et al. [14]^b^	India	Hospital/Tertiary	Synchronous	Follow-up	Cornea/Cataract	120 vs 120	41-80 (range)	45.8
Owsley et al. [15]	USA	Community/Optometry	Asynchronous	New/Screening	Glaucoma	500 (499)	58.1 ± 13.5	64.4
Appukumran et al. [16]	India	Remote/Mobile	Asynchronous	New/Screening	Medical Retina	12,157	NR	57.5
Mosenia et al. [17]^a^	USA	Hospital/Tertiary	Hybrid	Mixed	Glaucoma	123	63.9 ± 15.3	48.4
Koch et al. [18]	Brazil	Hospital/Tertiary	Synchronous	Mixed	General	161 (151)	46.0 ± 17.6	70.2
Ma et al. [19]	Canada	Hospital/Tertiary	Hybrid	New/Screening	Cornea/Anterior	1,535 (397)	54.9 ± 19.2	56.0
Iselin et al. [20]	Ireland	Hospital/Tertiary	Hybrid	Follow-up	Cornea/Cataract	88 (69)	NR	NR
Shah et al. [21]	India	Hospital/Tertiary	Asynchronous	Mixed	Medical Retina	104	53.2 ± 16.4	39.4
Juaristi et al. [22]^a^	Spain	Hospital/Tertiary	Asynchronous	Follow-up	Medical Retina	481	67.0 ± 13.9	54.2
Razak et al. [23]^b^	Malaysia	Community/Optometry	Synchronous	Follow-up	General	25 vs 25	33.8 ± 15.9	74.0
Gomel et al. [24]	Israel	Community	Asynchronous	New/Screening	Medical Retina/Gen.	124 (89)	79.8 ± 7.1	66.9
Hakim et al. [25]	UK	Hospital/Tertiary	Synchronous	Follow-up	General	84 (51)	62.4	64.7
Swystun et al. [26]	UK	Community/Optometry	Synchronous	New/Screening	Emergency	1106 (629)	57.0 (median)	45.0
Sieberer et al. [27]	UK	Hospital/Tertiary	Synchronous	New/Screening	Emergency	107 (104)	NR	NR
Conway et al. [28]	USA	Hospital/Tertiary	Synchronous	Follow-up	Neuro-ophth.	159	NR	NR
Huang et al. [29]	Singapore	Hospital/Tertiary	Asynchronous	Follow-up	Glaucoma	377 (214)	63.8 ± 11.6	67.3
Golash et al. [30]	UK	Hospital/Tertiary	Synchronous	Follow-up	Glaucoma	209 (120)	18-85 (range)	53.3
Bourdon et al. [31]^a^	France	Community/Primary	Synchronous	New/Screening	Emergency	1901 (176)	48.3 ± 14.5	76.0
Sharara et al. [32]	UK	Hospital/Tertiary	Sync & Async	Follow-up	Oculoplastics	100	39-93 (range)	48.0
Gerbutavicius et al. [33]	Germany	Community/Optometry	Synchronous	Mixed	General	29 (20)	59.3	55.2
Ali et al. [34]^b^	UK	Hospital/Tertiary	Asynchronous	Follow-up	Glaucoma	50 vs 55	70.9 ± 11.5	NR
Newman-Casey et al.[35]^b^	USA	Hospital/Tertiary	Synchronous	Follow-up	General	1720 (394 vs 521)	64.7 ± 16.8	58.7
Stafferi et al. [36]	Australia	Hospital/Tertiary	Synchronous	New/Screening	Paediatrics	211 (89)	NR	NR
Li et al. [37]	UK	Hospital/Tertiary	Synchronous	New/Screening	Emergency	568	43.0 (median)	54
Arntz et al. [38]	Chile	Hospital/Tertiary	Synchronous	New/Screening	General	291 (91)	38.0 ± 22.5	58.1
Alhumud et al. [39]	Saudi Arabia	Hospital/Tertiary	Asynchronous	New/Screening	Medical Retina	163	44.8	46.0
Valpuesta Martin et al. [40]	Spain	Remote/Mobile	Asynchronous	New/Screening	Medical Retina	114	68.7 (median)	41.2
Bastos de Carvalho et al. [41]	USA	Community/Optometry	Asynchronous	New/Screening	Medical Retina	13,923 (941)	54.9 ± 12.5	50.6
Modjtahedi et al. [42]	USA	Community/Optometry	Asynchronous	Follow-up	Glaucoma	225 (100)	58.1 ± 14.2	63.1
Host et al. [43]	Australia	Remote/Mobile	Hybrid	Mixed	General	137 (109)	64.6 ± 17.6	45.0
Maa et al. [44]	USA	Community/Optometry	Asynchronous	New/Screening	General	2,690 (1,126)	62.8 ± 12.1	9.2
Tan et al. [45]	Singapore	Remote/Mobile	Hybrid	New/Screening	General	30	62.0 (median)	56.7
Paul et al. [46]	India	Remote/Mobile	Hybrid	New/Screening	General	348	53.4 ± 12.9	44.5
Kumari et al. [47]	India	Remote/Mobile	Asynchronous	New/Screening	Medical Retina	132	53.0	41.0
Tuulonen et al. [48]^b^	Finland	Remote/Mobile	Hybrid	Follow-up	Glaucoma	29 vs 41	64.0 ± 14.0	66.0

Age is reported as mean ± SD if available.

*N* sample size, *SD* standard deviation, *NR* not reported, *survey n* the number of participants who filled the survey.

^a^Study assessed acceptability outcomes only (satisfaction data not reported).

^b^Comparative study (Tele-ophthalmology vs. In-Person care).

### Tele-ophthalmology vs in-person care satisfaction rates

**Fig. 2:**
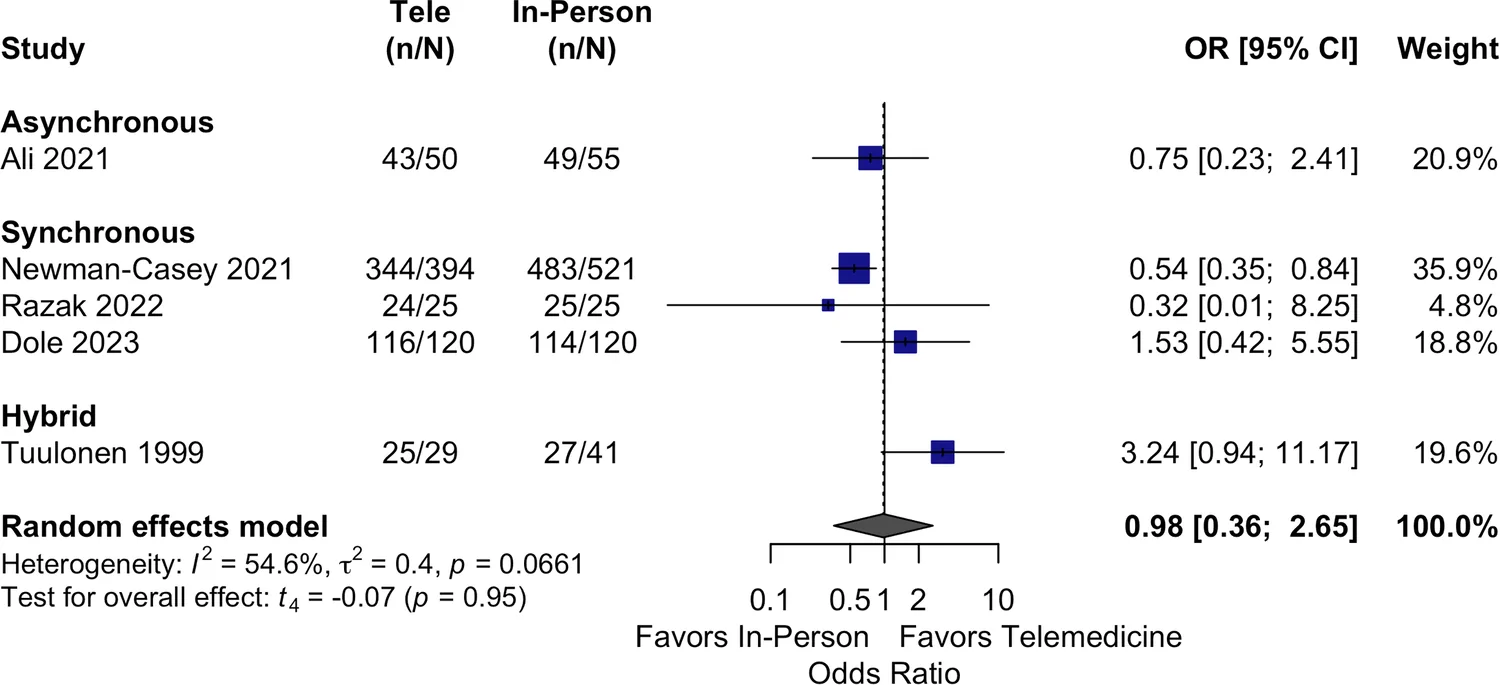
Forest plot for comparative meta-analysis. A forest plot for patient satisfaction rates comparison of in-person vs telemedicine eye care. Five studies were included with moderate heterogeneity between studies demonstrating a comparable satisfaction rate between both modalities of care.

Five studies comparing telemedicine (*n* = 618) and in-person visits (*n* = 762) showed no significant difference in satisfaction rates (OR = 0.98, 95% CI: 0.36–2.65, *t* = -0.07, *p* = 0.95), (Fig. 2). There was non-significant moderate between-study heterogeneity.

Due to the limited number of comparative studies, we did not run subgroup analysis. Instead, we employed the leave-one-out sensitivity analysis that showed satisfaction rates reported by Tuulonen et al. and Newman-Casey et al. were the main causes of the observed heterogeneity due to their opposing main effects.

### Tele-ophthalmology satisfaction rates

**Fig. 3:**
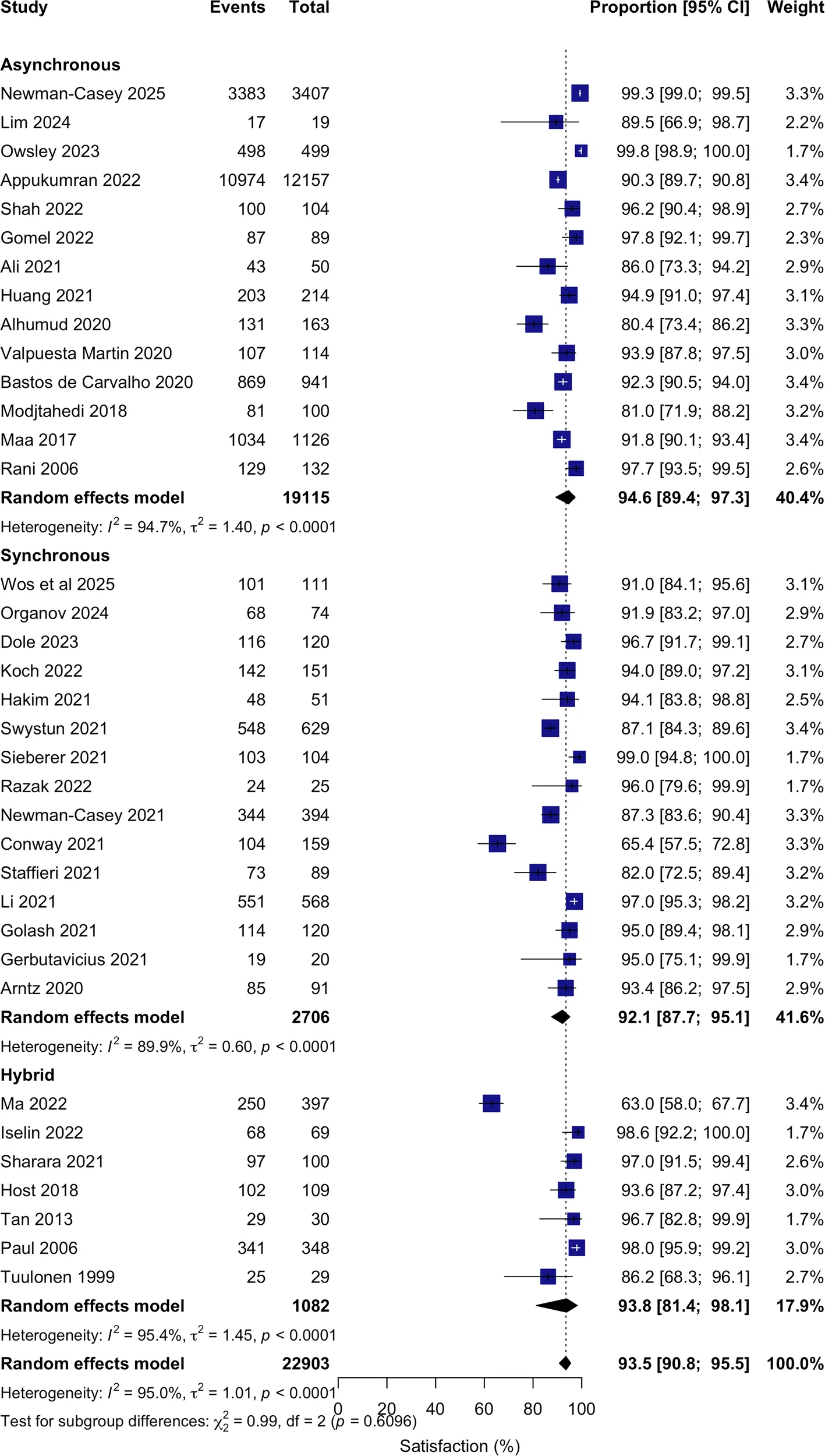
Forest plot for satisfaction rates in tele-ophthalmology. A forest plot presents patient satisfaction rates in telemedicine eye care across 36 studies, including 22,903 participants and 20,983 events (those favoured telemedicine). The overall satisfaction rate in tele-ophthalmology was 93.5%. A significant between-study heterogeneity was observed.

The pooled satisfaction proportion across 36 studies (*N* = 22,903) was 93.5% (95% CI: 90.8–95.5), (Fig. 3). Although the overall rate was high, there was significant substantial between-study variance (*Q* = 693.4*, df* = 35*, p* < 0.001; *I*^2^ = 95.0%). This prompted further sub-group analysis to investigate influencing factors.

Here, satisfaction rates remained consistent across different eye care delivery models [(${\chi }^{2}=0.99,{df}=2,{p}=0.61$), asynchronous (94.6%), synchronous (92.1%) and hybrid (93.8%)]. Comparable satisfaction rates were also observed across service settings, namely community/optometry practices 95.8% [88.0; 98.6], hospital/tertiary care 91.9% [87.3; 95.0] vs remote/mobile 94.2% [89.5; 96.8] eye services, ${\chi }^{2}=2.0,{df}=2,{p}=0.4$. Similarly, satisfaction rates were also comparable between newly examined and routinely followed-up participants (94.4% [90.3; 96.8] vs 91.5% [85.1; 95.3]), respectively, ${\chi }^{2}=1.6,{df}=2,{p}=0.45$. Finally, we reported high comparable satisfaction rates 94.4% [90.2; 96.9] among patients attending the three most common telemedicine eye specialties (Supplementary Fig. 1): Emergency 94.7% [75.1; 99.1], glaucoma 95.7% [75.5; 99.4] and medical retina 92.9% [87.3; 96.1], ${\chi }^{2}=0.59,{df}=2,{p}=0.75$.

Additionally, the chronological period of telemedicine implementation did not significantly alter the satisfaction rates [Mixed effect meta-regression (F (1, 34) = 0.0005, *p* = 0.98].

The pooled satisfaction rate across the 36 studies was robust as confirmed by the leave-one-out analysis that showed consistent range of overall rates between from 90.17% to 95.66% across iterations.

We assessed publication bias using Egger’s linear regression and visual inspection of funnel plots (Supplementary Fig. 2) that showed no significant evidence of bias (*t* = 1.28*, df* = 34*, p* = 0.21).

### Recommendation and intention to reuse

**Fig. 4:**
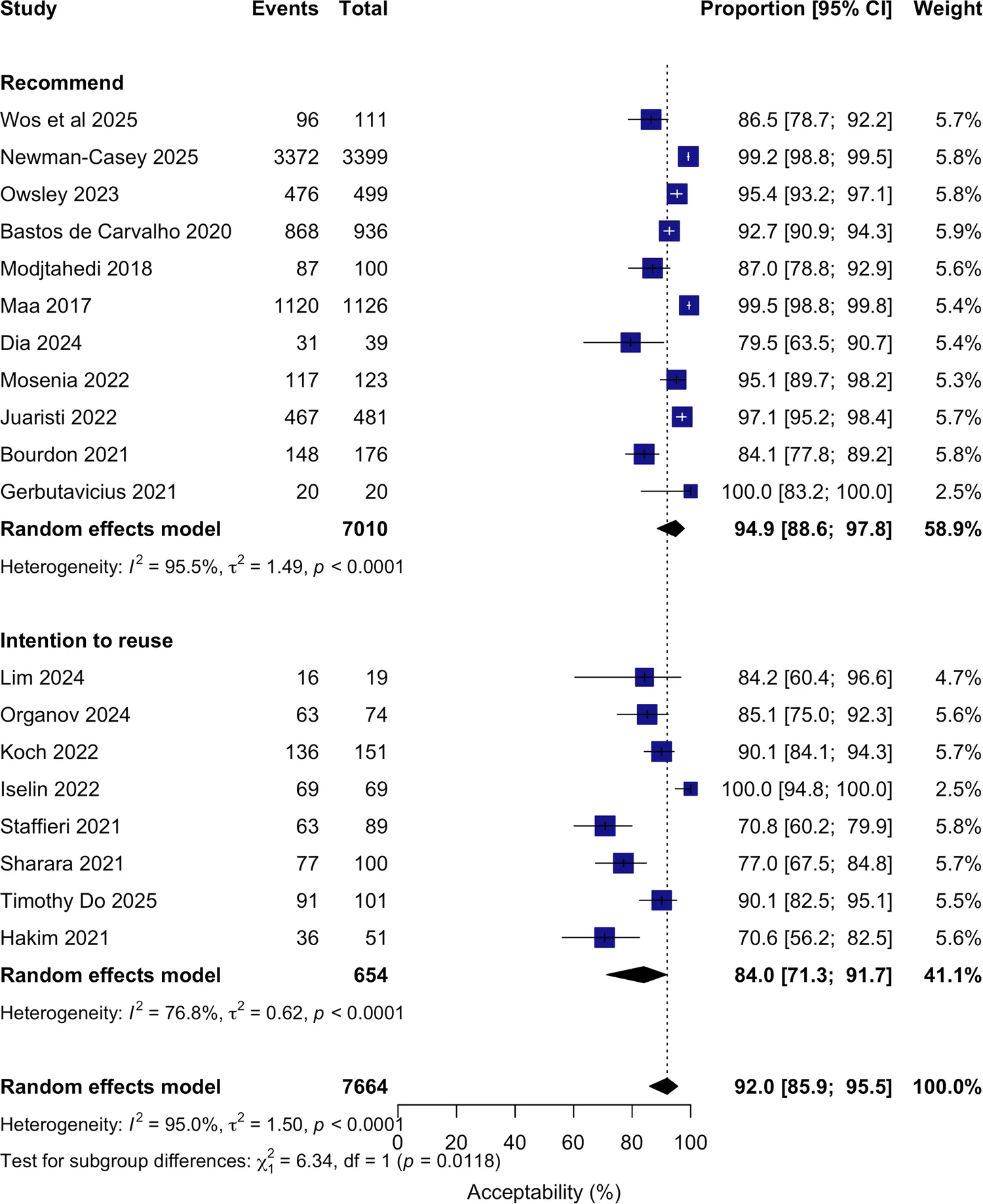
Forest plot for acceptability of tele-ophthalmology. The plot depicts rates for 19 studies involving 7664 participants. The overall rate of acceptability was 92.0% with significant between-study heterogeneity. While participants intended to recommend the use of such technology (94.9%), they showed lower intention rates of reuse (84.0%).

Nineteen studies (*n* = 7664 participants) reported data on service recommendation and/or future reuse (Fig. 4). The overall acceptability rate of tele-ophthalmology was 92.0% [85.9; 95.5]. However, there was a significant discrepancy in patient attitudes (${\chi }^{2}=6.34,{df}=1,{p}=0.01$, and *I*^2^ statistic = 95.0%). While 94.9% [88.6; 97.8] of participants were willing to recommend the service, the intention to reuse tele-ophthalmology was notably lower at 84.0% [71.3; 91.7]. There was no publication bias as per Egger’s test (*t* = −0.19*, df* = 17*, p* = 0.85).

### Methodological assessment and risk of bias

Supplementary Fig. 3 presents the overall assessment of studies using the JBI critical appraisal list. In both tele-ophthalmology studies (Supplementary Fig. 3A) and comparative studies (Supplementary Fig. 3B), the selection of participants and the outcome measurement showed low risk of bias. In tele-ophthalmology studies, the majority of studies reached a low-risk threshold for methodological rigor. Some comparative studies showed some concerns due to the identification and handling of confounders.

## Discussion

In this meta-analysis, including 5 comparative studies, we found that patient satisfaction rates in telemedicine were comparable to in-person eye care (OR = 0.98; 95% CI = 0.36; 2.65). We also found, analysing 36 studies (*N* = 22,903), that the patient satisfaction rate in telemedicine was remarkably high at 93.5% (95% CI = 90.8; 95.5). This evidence supports tele-ophthalmology as a highly acceptable modality as an alternative or complementary to standard eye care. To our knowledge, this is the first systematic grouping of patient satisfaction rates in tele-ophthalmology. These findings align with other medical subspecialties reporting comparable satisfaction rates, such as orthopaedics (OR = 0.82) [49], and a parallel systematic review of telemedicine during the COVID-19 era that reported overall satisfaction rates of 81% [50].

An important addition of our findings to the body of literature was the comparable satisfaction rates between hybrid (94%), synchronous (92%) and asynchronous (95%) models of tele-ophthalmology service. This mitigates potential concerns of lack of direct communications and patient acceptability when employing store-and-forward models of eye care. In contrast, a systematic review of telepsychiatry reported higher satisfaction rates for synchronous (97%) than asynchronous consultations (84%), which might be attributed to patients’ concerns with delayed feedback in the latter model of care [51]. This might support further expansion of scalable and cost-effective asynchronous services in resource-limited countries and regions particularly if combined with faster feedback to participants.

While the modality of care does not diminish overall satisfaction, long-term adoption faces distinct barriers. Here, we identified a significant discrepancy between patients’ willingness to recommend telemedicine services (95%) and their intentions for future use (84%). This gap shows that even though patients prefer telemedicine as a community service, they might frequently default back to standard in-person care due to different factors. These might include the underlying disease progression and structural barriers related to digital literacy and/or demographics. Recent literature indicates that digital exclusion factors such as comfort with digital technologies and older ages are among the common barriers to the use of telemedicine [52–54].

A key limitation of this review was the substantial heterogeneity between studies in the pooled proportion analysis (36 studies, *I*^2^ = 95%). We performed a subgroup analysis on modalities and service settings, still found high variance between studies. Across tele-health patient satisfaction reviews, it seems that heterogeneity is a common feature and is repeatedly attributed to factors such as population, clinical context, and modality employed. A systematic review of patient satisfaction in tele-health reported that modality might be a potential moderator of such heterogeneity, yet it does not eliminate it [50]. Possibly, the lack of standardised satisfaction assessment instruments and definitions makes it difficult to consistently measure satisfaction as a uniform and multidimensional construct.

This review has other limitations. First, the comparative meta-analysis was not generalisable due to the small number of studies included. Second, pooling rates across non-standardised and different questionnaire scales introduces measurement bias. Finally, satisfaction rates might have inflated during the COVID-19 pandemic due to psychological and access constraints. However, we found that the reported rates are not driven by time trends as evidenced by our meta-regression, which showed that implementation time was not a significant influencer.

Tele-ophthalmology achieves high satisfaction rates that are comparable to in-person eye care. These findings did not depend on whether the delivery mode was synchronous or asynchronous. We identified a preference gap between willingness to recommend and future use, which highlights a need to address barriers such as digital literacy and disease specific requirements in order to efficiently utilise telemedicine services. Due to the high heterogeneity reported, it is advisable to standardise survey instruments and definitions in order to inform policy makers and optimise telemedicine strategies.

Supplemental material is available at Eye’s website.

## Supplementary information

Supplementary Figure 1. A forest plot for satisfaction rates across three common tele-ophthalmology modalities.

Supplementary figure 2. Funnel plot asymmetry.

Supplementary Figure 3. Methodological quality assessment.

Supplemental Material.

## Data Availability

The data used in this meta-analysis were extracted from previously published studies, which are cited within the article. No new data were generated.
