# Supplementary material for: Patient satisfaction in tele-ophthalmology: systematic review and meta-analysis
Source: Eye (Lond). 2026 Sep 7;40(16):2499–506. doi: 10.1038/s41433-026-04831-4 (PMC13754922; doi:10.1038/s41433-026-04831-4)
Supplement: Supplementary file 1 [file 41433_2026_4831_MOESM4_ESM.docx]

**Supplemental Material**

Supplementary Figure 1. A forest plot for satisfaction rates across three common tele-ophthalmology modalities.

A forest plot presents patient satisfaction rates in telemedicine eye care across 18 studies, including 19441 participants. A high and comparable satisfaction rates were reported with emergency eye services (94.7%), glaucoma (95.7%) and medical retina clinics (92.9%).

Supplementary figure 2. Funnel plot asymmetry.

The plot depicts satisfaction proportions (logit-transformed) on x-axis vs the standard error on y-axis for 36 studies for the assessment of publication bias for tele-ophthalmology satisfaction rates. The central and both diagonal lines depict the pooled rate and 95% CI, respectively. Studies appear to distribute equally across both sides of the central line confirming non-significant Egger’s test for asymmetry (p = 0.32), hence absence of publication bias. Horizontal dispersion of studies corroborates the high level of heterogeneity.

Supplementary Figure 3. Methodological quality assessment.

Methodological rigor using JBI Critical Appraisal Checklists for A) Tele-ophthalmology studies (n =36) and B) Comparative studies (n = 5). Most studies showed robust methodology but comparative studies did not sufficiently address potential confounders such as age, disease severity or access to care.
